# Association between pulmonary function and peak oxygen uptake in elderly: the Generation 100 study

**DOI:** 10.1186/s12931-015-0317-0

**Published:** 2015-12-30

**Authors:** Erlend Hassel, Dorthe Stensvold, Thomas Halvorsen, Ulrik Wisløff, Arnulf Langhammer, Sigurd Steinshamn

**Affiliations:** K.G. Jebsen Center of Exercise in Medicine at Department of Circulation and Medical Imaging, Faculty of Medicine, Norwegian University of Science and Technology, Trondheim, Norway; SINTEF Technology and Society, Department of Health Research, PO Box 4760, Sluppen, N 7465 Trondheim, Norway; Department of Public Health and General Practice, Faculty of Medicine, Norwegian University of Science and Technology, Trondheim, Norway; Clinic of Thoracic and Occupational Medicine, St. Olavs Hospital, Trondheim University Hospital, PO Box 3250, Sluppen, 7006 Trondheim, Norway

**Keywords:** Exercise and lung function, Respiratory physiology, Ageing

## Abstract

**Background:**

Although reduced function of the respiratory system limits peak oxygen uptake in diseases affecting the lungs or airways, the healthy respiratory system is thought to have a spare capacity for oxygen transport and uptake, and is not considered a limiting factor for peak oxygen uptake in healthy people. However, lung function declines with age and could theoretically limit peak oxygen uptake in elderly. We examined the association between peak oxygen uptake and lung function indices in an elderly population with the hypothesis that lung function indices would be associated with VO_2peak_ up to a threshold value situated above the lower limits of normal lung function for our population.

**Methods:**

Spirometry, gas diffusion tests and incremental work tests were performed in 1443 subjects (714 women) aged 69–77 years. Association between lung function indices and peak oxygen uptake was studied with hockey-stick regression.

**Results:**

Forced expiratory volume in 1 s (FEV_1_) had a positive association with peak oxygen uptake up to, but not above, a threshold value of 2.86 l for men, and 2.13 l for women (lower limit of normal 2.73 and 1.77 l respectively). A corresponding threshold was found for diffusing capacity of the lung for carbon monoxide (D_LCO_) for men at 9.18 mmol/min/kPa (lower limit of normal 6.84 mmol/min/kPa). D_LCO_ for women and D_LCO_ divided by alveolar volume (D_LCO_/VA) for both sexes had a significant linear relationship to VO_2peak_ (*p* < 0.05), but no significant threshold value was found in these associations.

**Conclusions:**

Threshold values for FEV_1_ for both sexes and D_LCO_ for men were identified. These lung function indices had a positive association with VO_2peak_ up to these threshold values, but not above. The identified threshold values were above lower limits of normal for FEV_1_ and D_LCO_.

**Electronic supplementary material:**

The online version of this article (doi:10.1186/s12931-015-0317-0) contains supplementary material, which is available to authorized users.

## Background

It is generally accepted that maximal cardiac output is the principal limiting factor for peak oxygen uptake (VO_2peak_) in healthy individuals exercising at sea-level. By contrast the respiratory system has been considered to be structurally overbuilt both with respect to dynamic lung function and diffusion capacity, and is therefore believed not to restrict oxygen uptake even during maximal exercise in non-endurance athletes [1, 2]. Reduced lung function limits VO_2peak_ in patients with pulmonary disease [3–7], but the association with VO_2peak_ for lung function indices within the normal range is little studied.

In healthy ageing there is a steady decline of dynamic lung volumes. Expiratory flow is reduced and the flow-volume curve may resemble what is found in patients with chronic obstructive pulmonary disease [8]. The capacity of the lung for gas diffusion is also reduced with age [9]. Elderly have an increased dead space to tidal volume ventilation ratio compared to younger individuals and develop a marked alveolar to arterial oxygen gradient during exercise [10]. The spare capacity of the respiratory system is possibly reduced in elderly compared to middle-aged and young people, and both dynamic lung function and the diffusion capacity of the lungs may therefore be associated with reduced oxygen uptake even in healthy elderly people. This has previously not been examined in population based studies.

Given that the healthy respiratory system is overbuilt with respect to requirements for oxygen uptake, one would observe an association between lung function and VO_2peak_ only when the capacity of the respiratory system is reduced below a threshold value which is below the lower limits of normal lung function. However, if the spare capacity of lung function is reduced with increasing age, this threshold may be found within the range of normal lung function in an elderly population, in that case suggesting that VO_2peak_ may be limited by lung function in many healthy elderly. The present study examined the association between VO_2peak_ and parameters from spirometry and gas diffusion tests in a large population based sample of elderly men and women.

We wanted to investigate if lung function indices were associated with VO_2peak_ up to a threshold value above which further increase in lung function would not be associated with increasing VO2peak. If a threshold value were identified, we hypothesized that this threshold would be above the LLN (lower limits of normal) for lung function, i.e. within the normal range of lung function for this population.

## Methods

### Study population

The ongoing Generation 100 Study invited all inhabitants in the city of Trondheim, Norway, born between 01 January 1936 and 31 December 1942 (*n* = 6966) to a randomized controlled trial on the effect of exercise intervention on morbidity and mortality in an ageing population [11]. Study subjects were included from August 2012 to June 2013 giving a study population aged 69–77 years. The present study includes baseline data of participants that were able to perform exercise testing and training. Participants with chronic communicable infectious diseases, dementia, uncontrolled hypertension, heart failure, cardiomyopathy, severe arrhythmia, participating in other studies conflicting with Generation 100, or with conditions or test results indicating that testing or training could be unsafe were excluded. The Generation 100 Study and the present sub-study were approved by the Regional Committee for Medical Research Ethics (REK 2012/381 B) and all participants gave written informed consents.

### Examinations

A symptom-limited incremental work test on a treadmill was performed to measure VO_2peak_ using MetaMax II (Cortex, Leipzig, Germany) or Oxycon Pro (Erich Jaeger, Hoechberg, Germany) as previously described [12]. Participants not able to perform the test on a treadmill due to poor balance or other reasons performed the test on a cycle ergometer. Load was increased 1 km/h or 2 % inclination on the treadmill approximately every 90 s until exhaustion, on the bicycle load was increased 10 W every 30 s. Testing of participants with suspected or previously diagnosed heart disease was supervised by a trained physician [13]. For simplicity the term VO_2peak_ is used throughout this paper, even when referring to work by others that have reported maximum oxygen uptake. The participants reported average physical activity by answering three questions covering frequency, intensity and duration of physical activity. The answers were given different weights and a physical activity index was calculated as previously described [14].

Spirometry and gas diffusion test were performed with Sensormedics Vmax22 Encore (CareFusion, San Diego, USA) in accordance with the American Thoracic Society/European Respiratory Society recommendations [15, 16]. The spirometer was calibrated daily. The study participants performed up to a maximum of eight spirometry trials until forced expiratory volume at 1 s (FEV_1_) and forced vital capacity (FVC) showed <150 ml variation between the two highest results. The highest values for vital capacity (forced expiratory or inspiratory) and FEV_1_ were recorded. No reversibility test was performed. For the day of testing, participants were instructed to continue any medication as usual, including anti-obstructive treatment. Predicted values and lower limit of normal (LLN) were calculated from Norwegian reference equations using age, sex and height [17].

Diffusing capacity of the lung for carbon monoxide (D_LCO_) was measured by the single breath-method using a gas mixture containing 0.3 % carbon monoxide and 0.3 % methane. Estimated alveolar volume (VA) was calculated based on the dilution of methane from inspired to expired gas. The procedure was repeated with at least 4 min between each trial up to a maximum of 4 trials, until two tests of acceptable quality show D_LCO_ within 1 mmol/min/kPa or within 10 % of the highest value and the mean of these values were recorded. Predicted values were calculated using equations developed for a comparable age group [18].

The spirometry and D_LCO_-test were administered by a pulmonary care nurse or medical doctor and all measurements were quality controlled.

Body-fat percentage was measured with bioelectric impedance analysis (Inbody 720, Seoul, South Korea). In addition to the clinical tests, the participants filled out health-related questionnaires.

### Statistics

All analyses were stratified by sex. A statistical procedure called hockey-stick regression was applied [19]. It assumes that the effect of variable x is best fitted by two continuous linear functions; the slopes of both lines and also the change-point where the two lines meet are estimated. The resulting combined fitted line has one slope up to a change-point on the x-axis and a different slope after the change-point. Hockey-stick regressions with VO_2peak_ (ml/min/kg) as the dependent variable were performed; FEV_1_, D_LCO_ and D_LCO_ adjusted for alveolar volume (D_LCO_/VA) were tested as variables with change-points in separate models. For comparison linear and curvilinear models were also fitted. In the linear models FEV_1_, D_LCO_ and D_LCO_/VA were added as linear variables; and in the curvilinear models these measurements were added as both linear and squared variables. The analyses were controlled for age, physical activity index, resting heart rate and body-fat percentage, smoking status and self-reported heart disease. Covariates were chosen based on previous prediction models for VO_2peak_ [14, 20]; self-reported heart disease was also added due to potential confounding in this age group. Control variables were continuous linear variables, except for current smoking (coded 1 or 0) and self-reported heart disease coded 1 for self-reported history of myocardial infarction, angina pectoris or atrial fibrillation, otherwise 0. Chow test with null-hypothesis that intercept and coefficient are equal before and after change point were used to test for structural break at change-point, and f-test were used to compare hockey-stick and curvilinear models to linear models. All calculations were performed with Stata 13.1 (StataCorp, Texas, USA); hockey-stick regressions were modelled with the “nl hockey” function.

## Results

A total number of 1567 VO_2peak_-tests were completed; of these 47 on a cycle ergometer. Forty cases were omitted from the analyses due to submaximal effort (maximal self-reported value <15 on the 6-20 Borg scale); and 84 were omitted due to missing physical activity score, smoking status, body-fat percentage or valid spirometry data. Descriptive statistics for the 1443 participants included in the analyses are shown in Table 1, histograms showing the distribution of VO_2peak_, FEV_1_ and D_LCO_ and D_LCO_/VA are shown in Additional file 1.

**Table 1:** Descriptive statistics

	Males *n* = 729	Females *n* = 714
Age	72.8 ± 2.1	72.9 ± 2.1
Height (cm)	176.9 ± 5.9	163.4 ± 5.3
Weight (kg)	82.5 ± 11.3	68.4 ± 10.9
Body mass index (kg/cm^2^)	26.3 ± 3.2	25.6 ± 3.8
Body-fat percentage	25.5 ± 6.3	34.7 ± 7.0
Resting heart rate (beats per minute)	62.7 ± 11.2	66.8 ± 10.0
Physical activity index	9.3 ± 8.6	8.0 ± 8.7
Self-reported heart disease (% yes) (MI, angina or atrial fibrillation)	18.6 %	5.5 %
Self-reported lung disease (% yes) (asthma, chronic bronchitis, emphysema, chronic obstructive pulmonary disease)	10.8 %	12.5 %
Smoking status:		
Never	41.5 %	54.2 %
Former	50.5 %	37.4 %
Current	7.9 %	8.4 %
Pack years	8.2 ± 14.2	4.9 ± 10.5
FEV_1_ (litres)	3.13 ± 0.60	2.24 ± 0.37
FEV_1_ % of predicted	94.0 ± 16.7	102.4 ± 15.9
FEV_1_/FVC (%)	72.1 ± 8.1	73.4 ± 6.6
D_LCO_ (mmol/min/kPa)	9.04 ± 1.68	6.73 ± 1.14
D_LCO_ % of predicted	93.5 ± 16.0	86.3 ± 13.5
D_LCO_/VA (mmol/min/kPa/l)	1.37 ± 0.22	1.40 ± 0.20
D_LCO_/VA % of predicted	93.7 ± 18.2	84.8 ± 11.9
Peak oxygen uptake (ml/min/kg)	31.3 ± 6.7	26.1 ± 5.0
Respiratory Exchange Ratio at peak work	1.14 ± 0.09	1.10 ± 0.09
Peak heart rate (bpm)	156.7 ± 17.7	156.5 ± 15.9
Ventilation at peak exercise (litres)	96.4 ± 21.2	61.2 ± 12.5

Definition of abbreviations: MI = myocardial infarction; FEV_1_ = forced expiratory volume in 1 s; FEV_1_/FVC = FEV_1_ divided by forced vital capacity; D_LCO_ – diffusing capacity of the lung for carbon monoxide; D_LCO_/VA – D_LCO_ corrected for estimated alveolar volume. Values are given as percentage or mean ± standard deviation

**Fig. 1:**
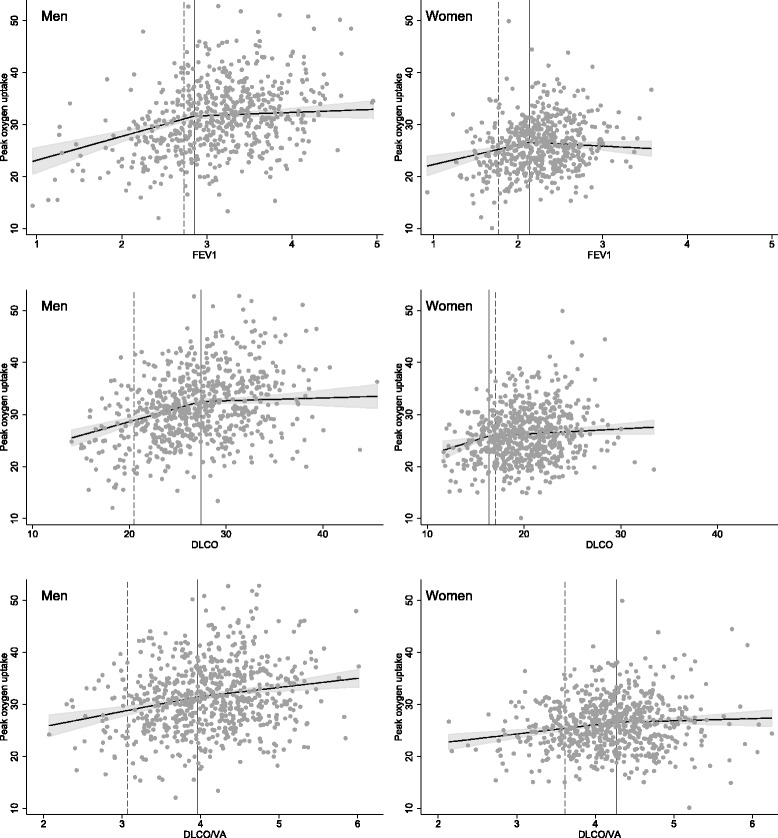
Predicted effect plots for hockey-stick models. In separate models forced expiratory volume in 1 s (FEV_1_) in litres, diffusing capacity of the lung for carbon monoxide (D_LCO_) in mmol/min/kPa and D_LCO_ corrected for estimated alveolar volume (D_LCO_/VA) in mmol/min/kPa/l were added as variables with change points. Dependent variable is peak oxygen uptake in ml/min/kg. All models included age, self-reported physical activity, resting heart rate, body-fat percentage, self-reported history of heart disease and current smoking status as control variables. To illustrate the isolated effect of the lung function parameters, control variables were set to sample mean for these plots. Vertical lines mark change-points; dashed lines mark lower limit of normal; grey fields mark 95 % confidence interval for slopes; dots mark observations. Change-points were significant (*p* < 0.5) for FEV_1_ for both sexes and for D_LCO_ for men, but not for D_LCO_ for women and D_LCO_/VA for neither sex

Significant change-points in the association with VO_2peak_ were found for FEV_1_ for men at 2.86 l (LLN = 2.73 l) and for women at 2.13 l (LLN = 1.77 l) (Table 2). The association between FEV_1_ and VO_2peak_ was positive up to these values, but not above. The change-points correspond to the 31st and 28th percentile of measured FEV_1_, for men and women respectively. A change-point was also found in the association between D_LCO_ and VO_2peak_ for men at 9.18 mmol/min/kPa (LLN = 6.84 mmol/min/kPa), corresponding to the 54th percentile. No significant change-point in the association between D_LCO_ and VO_2peak_ was found for women. Furthermore, no significant change-points were found in the association between D_LCO_/VA and VO_2peak_ for either sex. Predicted effect plots for these hockey-stick regression models are shown in Fig. 1.

**Table 2:** Change-point regression for association with peak oxygen uptake (ml O_2_/min/kg)

	Change point (95 % CI)	Left slope (95 % CI)	Right slope (95 % CI)
Men:			
FEV_1_ (*n* = 729)	2.86 litres* (2.54, 3.17)	4.58† (2.85, 6.31)	0.60 (-0.56, 1.75)
D_LCO_ (*n* = 712)	9.18 mmol/min/kPa* (8.32, 10.05)	1.57† (1.06, 2.07)	0.16 (-0.37, 0.68)
D_LCO_/VA (*n* = 712)	1.36 mmol/min/kPa/l (0.99, 1.74)	8.54† (4.32, 12.75)	4.99† (0.89, 9.08)
Women:			
FEV_1_ (*n* = 714)	2.13 litres * (1.93, 2.34)	3.76† (1.60, 5.92)	−0.84 (-2.22, 0.55)
D_LCO_ (*n* = 697)	5.50 mmol/min/kPa (4.45, 6.56)	1.54 (-0.04, 3.12)	0.31 (-0.01, 0.62)
D_LCO_/VA (*n* = 697)	1.43 mmol/min/kPa/l (1.18, 1.68)	5.14† (2.37, 7.91)	1.25 (-2.30, 4.80)

Definition of abbreviations: FEV_1_ = forced expiratory volume in 1 s; D_LCO_ – diffusing capacity of the lung for carbon monoxide; D_LCO_/VA – D_LCO_ corrected for estimated alveolar volume. Non-linear “Hockey-stick” regressions for the associations between pulmonary function variables and peak oxygen uptake. * *p* < 0.01 for hockey-stick model vs. linear model (f-test), † *p* < 0.01 for slope being different from 0

Explained variance (adjusted R^2^) for VO_2peak_ from the hockey stick models were compared to corresponding linear and curvilinear models (Table 3). For the association between FEV_1_ and VO_2peak_ the hockey-stick model gave higher R^2^-values than the linear and curvilinear models (R^2^ = 0.433 (hockey-stick) vs. 0.423 (linear) and 0.429 (curvilinear) for men, and R^2^ = 0.460 (hockey-stick) vs. 0.452 (linear) and 0.456 (curvilinear) for women), supporting the choice of statistical model for this relationship. For D_LCO_ the hockey-stick model was significantly better than the linear model for men (R^2^ = 0.450 vs. 0.440), but not for women (R^2^ = 0.470 vs. 0.469); and for both sexes R^2^-values from hockey-stick model were similar to R^2^ from curvilinear models (Table 3). For D_LCO_ for women and for D_LCO_/VA for either sex, neither the hockey stick model nor the curvilinear model was significantly better than the linear model, suggesting linear relationships. Full descriptions of models are presented in Additional file 2.

**Table 3:** Explained variance of VO_2peak_ (Adjusted R^2^) of models with and without change points

Added variable	Hockey-stick model	Linear model	Curvilinear model
Men:			
FEV_1_	0.433*	0.423	0.429*
D_LCO_	0.450*	0.440	0.451*
D_LCO_/VA	0.440	0.441	0.441
Women:			
FEV_1_	0.460*	0.452	0.456*
D_LCO_	0.470	0.469	0.470
D_LCO_/VA	0.477	0.476	0.477

Definition of abbreviations: VO_2peak_ = peak oxygen uptake; FEV_1_ = forced expiratory volume in 1 s; D_LCO_ – diffusing capacity of the lung for carbon monoxide; D_LCO_/VA – D_LCO_ corrected for estimated alveolar volume. Resulting explained variance (Adjusted R^2^) of regression models with peak oxygen uptake as dependent variable with variable in first column added either as a variable with change point, as a linear variable or as a curvilinear variable (both variable and variable^2^). Age, self-reported physical activity, resting heart rate, body-fat percentage, self-reported history of heart disease and current smoking status are control variables in all models. *P-value < 0.05 for R^2^-change compared to linear model (f-test). Adjusted R^2^ for VO_2peak_ in a multiple linear model without pulmonary function measurements, including only age, physical activity index, resting heart rate, body-fat percentage, smoking status and heart disease history were 0.393 for men and 0.448 for women

To evaluate the effects of self-reported physical activity on the identified change-points, sub-group analyses were performed separately on those reporting high physical activity (physical activity index > = 15) and those reporting low activity (physical activity index < 15). A physical activity index score of 15 corresponds to reporting exercise once a week with high intensity and duration >30 min; or exercise 2–3 times per week with moderate intensity and duration >30 min; or exercise almost every day of moderate intensity and duration <30 min. This cut-point classifies 50.2 % of men and 44.7 % of women as reporting high physical activity. For men change-points for FEV_1_ were 2.90 l for those reporting high physical activity (LLN = 2.75 l) and 2.68 l for those reporting low activity (LLN = 2.73 l); the corresponding change-point localizations for D_LCO_ were 11.0 mmol/min/kPa for high activity (LLN = 6.82 mmol/min/kPa) and 7.13 mmol/min/kPa for low activity (LLN = 6.85 mmol/min/kPa). For corresponding sub-groups of women the change-point values for FEV_1_ were 2.13 l for high activity (LLN = 1.78 l) and 2.12 l for low activity (LLN = 1.77 l).

To assess the effect of lung disease in our analyses, self-reported pulmonary disease (history of asthma, chronic bronchitis, emphysema or chronic obstructive pulmonary disease) were tested as an independent variable in the regression analyses. Self-reported pulmonary disease was not a significant predictor for VO_2peak_ in the hockey-stick model for FEV_1_ for either sex, and did not increase R^2^ or affect the identified threshold value. In the hockey-stick model for D_LCO_ for men, self-reported pulmonary disease was a significant predictor for VO_2peak_, but had only a minimal effect on the value, which decrease from 9.18 to 9.07 mmol/min/kPa.

## Discussion

In this large population sample we have found evidence for a change-point in the association with VO_2peak_ for FEV_1_ and D_LCO_ in men and FEV_1_ in women. The associations between these variables and VO_2peak_ were significant up to the change-points, but no significant associations were found above these values. This suggests that there may be a physiologic threshold for these lung function parameters, above which the lung function does not limit VO_2peak_. The identified threshold values are within the normal limits of lung function for this age group, which may suggest that lung function can be a limiting factor for maximal physical performance for many healthy elderly. A linear association with VO_2peak_ was found for D_LCO_ for women and D_LCO_/VA for both sexes, but no significant threshold was found for these parameters.

The identified change-points for FEV_1_ equals 86 % of predicted for men and 97 % for women when age and height are set to sample means; and are higher than calculated LLN at 2.73 l for men and 1.77 l for women. Predicted values and LLN were calculated using relevant Norwegian reference equations [17]. If instead using the 2012 Global Lung Function Initiative equations [21] the change-points would be 93 % of predicted FEV_1_ for men and 99 % for women, so using the Norwegian reference values is the more conservative approach. The threshold for D_LCO_ for men corresponds to 95 % of predicted and is higher than LLN at 6.84 mmol/min/kPa. For the association between D_LCO_ and VO_2peak_ in women, neither the hockey-stick model nor the curvilinear model performed better than the multiple linear model; which may either be due to physiologic differences between sexes or to lack of statistical power to detect a levelling off of this relationship in women.

Several studies have shown that VO_2peak_ is reduced in patients with lung disease and reduced lung function [3–6], but there are few studies on the relationship between pulmonary function measurements and VO_2peak_ in healthy subjects. Babb et al. [22] reported a significant linear relationship between FEV_1_ and VO_2peak_ in a small sample of asymptomatic volunteers selected to have a wide range of FEV_1_ (*n* = 11, age = 58 ± 8 years). Johnson et al. [23, 24] studying a group of active healthy older subjects (*n* = 30, age = 70 ± 1 years) found a correlation between % of predicted D_LCO_ and VO_2peak_, and also showed that FEV_1_ were significantly higher in subjects with VO_2peak_ above the median compared to those with VO_2peak_ below the median. These studies had few participants and were not designed to study the association between lung function indices and VO_2peak_, and they do not allow general conclusions about this association. On the other hand, in an interventional study, Sue-Chu et al. [25] examined the effect of the bronchodilator salbutamol on VO_2peak_ in non-asthmatic highly-trained cross-country skiers with high VO_2peak_. These subjects did not improve the VO_2peak_ in spite of a significant improvement in FEV_1_, suggesting that even highly trained endurance athletes are not usually ventilatory limited with respect to VO_2peak_. A significant proportion of highly fit endurance athletes develops arterial hypoxemia during sub-maximal or maximal exercise [26]. This exercise-induced arterial hypoxemia limits VO_2peak_ and this limitation can be reversed by supplemental oxygen during exercise [27, 28], suggesting that diffusion capacity could be a limiting factor in these subjects.

Physical exercise improves the capacity of the cardiovascular and locomotor systems to transport and utilize oxygen, while no such effect is evident on the respiratory system. An improvement in the maximal function of the circulatory and muscular systems from exercise would therefore increase the demand on the respiratory system for oxygen transport, reducing any spare capacity of this system. We would therefore expect that the association between lung function indices and VO_2peak_ would be significant up to higher levels of FEV_1_ and D_LCO_ in an exercise-trained population compared to a more sedate one. This is in accordance with the sub-group analyses in our study showing change-points at higher levels of lung function in men with high self-reported physical activity compared to low self-reported activity. This effect is particularly pronounced for the association between D_LCO_ and VO_2peak_, suggesting that D_LCO_ may represent a limiting factor for exercise capacity for men in this age group. For women, the location of the change point in the association between FEV_1_ and VO_2peak_ is almost identical for those reporting high vs. low physical activity (2.13 vs. 2.12 l).

The findings in our study may be due to the age-related changes in the lung function of elderly people. With increasing age the lungs loose elastic recoil, the thorax wall gets stiffer and more restricted, respiratory muscle function is impaired, the alveolar surface area is reduced and there is increased ventilation-perfusion heterogeneity [29]. These changes cause expiratory flow limitation and reduced gas diffusion capacity in elderly compared to younger subjects. Even though there is a decline in the capacity for ventilation and gas exchange with age, there is also an age-related decline in the capacity of the other links of the oxygen uptake chain thus reducing the demands on the respiratory system. The margin between demand and capacity in the respiratory system decreases with age, but limitation of VO_2peak_ due to demands exceeding the capacity is thought to be rare [30]. Our findings of an association with VO_2peak_ for FEV_1_ and D_LCO_ in the lower reference area may suggest that a reduced capacity of the respiratory system may be limiting for VO_2peak_ for many elderly. The spare capacity of the lungs and airways seen in young healthy subjects may be fully eroded in many elderly, resulting in a limitation of VO_2peak_ and maximal work rate by the respiratory system for these individuals.

The main strength of the present study is the population-based design and high number of subjects with directly measured VO_2peak_ combined with spirometry and gas diffusion testing. To our knowledge, there are no other large studies examining the association between normal lung function and VO_2peak_. Even though known or plausible confounders were controlled for in our analyses, there might still be some residual confounding. Unrecognized pulmonary disease may contribute to residual confounding, but since pulmonary function is already included in the analyses, pulmonary disease would have to affect VO_2peak_ independently of pulmonary function if pulmonary disease were to be a confounder for this relationship. We cannot test whether unrecognized pulmonary disease affects the results, but we can test this for those with self-reported pulmonary disease. Self-reported pulmonary disease is a significant predictor of VO_2peak_ only when FEV_1_ is not included in the analyses, indicating that pulmonary disease is an up-stream variable of FEV_1_, and therefore not a relevant confounder in the analyses. Even though we have identified threshold levels in the associations between lung function indices and VO_2peak_ on the population level for our subjects, the thresholds cannot be assumed to apply to individual subjects. Neither can we conclude that a subject having lung function below the threshold values has a pulmonary limitation of VO_2peak_. The physiologic basis for the observed change-point in the association between lung function indices and VO_2peak_ may be age specific, and it cannot be assumed that the results in this study apply to younger or middle-aged populations. Ventilation-perfusion heterogeneity and widened alveolar-arterial oxygen pressure gradient are likely to be important factors for the limiting effect of the respiratory system on VO_2peak_; and measurements of blood oxygen concentration during exercise would likely have yielded further knowledge on this topic. Participants in this study were recruited to an exercise intervention study, and may be habitually physically active or at least more interested in exercise than the general population. This is a descriptive study with a cross-sectional design and it does not allow for conclusions on causality, and the results should therefore be interpreted with caution.

## Conclusions

We have found evidence of a threshold in the association between lung function measurements and VO_2peak_ in this elderly population. FEV_1_ for both sexes and for D_LCO_ for men were positively associated with VO_2peak_ only up to these threshold values. The identified threshold values are well within the normal range for these lung function parameters. D_LCO_ for women and D_LCO_/VA for both sexes were linearly associated with VO_2peak_. A possible explanation for our findings could be that lung function even within the normal range may be a limiting factor for maximal oxygen uptake for many elderly.

## Additional files

Additional file 1:
**Histograms showing the distribution of measured VO**_**2peak**_ **, FEV**_**1**_ **, D**_**LCO**_ **and D**_**LCO**_ **/VA by sex.** (JPG 547 kb) Additional file 2:
**Word document.** Full description of models. Description of statistical models with coefficients. (DOCX 22 kb)
