# Supplementary figures and images for: Association between pulmonary function and peak oxygen uptake in elderly: the Generation 100 study
Source: Respir Res. 2015 Dec 30;16:156. doi: 10.1186/s12931-015-0317-0 (PMC4699380; doi:10.1186/s12931-015-0317-0)

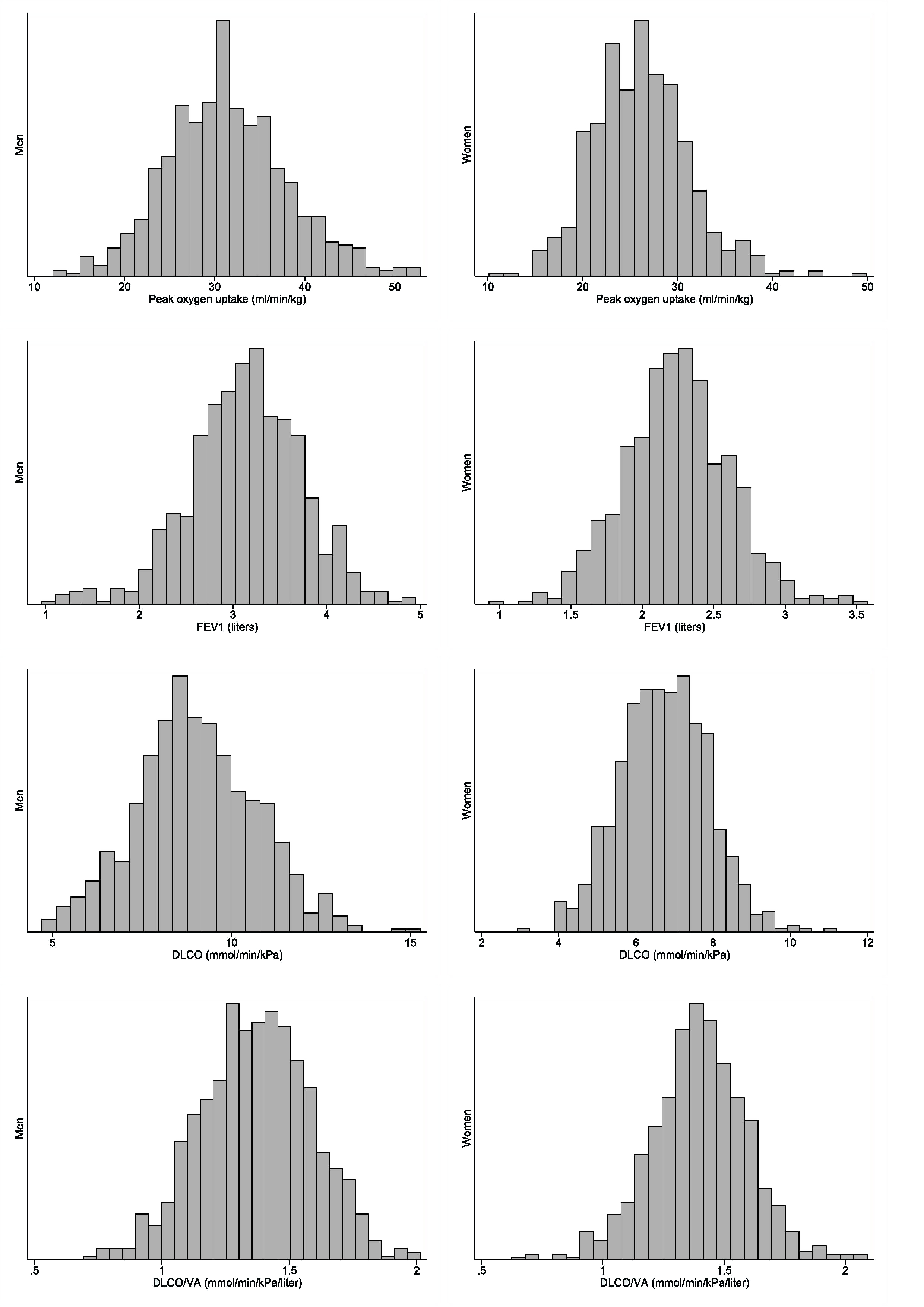

Supplement: Additional file 1: — Histograms showing the distribution of measured VO 2peak , FEV 1 , D LCO and D LCO /VA by sex. (JPG 547 kb) [file 12931_2015_317_MOESM1_ESM.jpg]
