# Supplementary material for: Association between pulmonary function and peak oxygen uptake in elderly: the Generation 100 study
Source: Respir Res. 2015 Dec 30;16:156. doi: 10.1186/s12931-015-0317-0 (PMC4699380; doi:10.1186/s12931-015-0317-0)
Supplement: Additional file 2: — Word document. Full description of models. Description of statistical models with coefficients. (DOCX 22 kb) [file 12931_2015_317_MOESM2_ESM.docx]

| Additional table 1. Hockey-stick regression models with VO_2peak_ (ml/min/kg) as dependent variable | | | | | | | |
| --- | --- | --- | --- | --- | --- | --- | --- |
|  | Men | | |  | Women | | |
|  | FEV_1_ | D_LCO_ | D_LCO_/VA |  | FEV_1_ | D_LCO_ | D_LCO_/VA |
| Constant | 54.72* | 44.18* | 47.82* |  | 52.63* | 46.67* | 49.42* |
| Change-point | 2.856 | 9.18 | 1.36 |  | 2.133 | 5.51 | 1.43 |
| Left slope | 4.585* | 1.567* | 8.538* |  | 3.759* | 1.542 | 5.143* |
| Right slope | 0.596 | 0.158 | 4.989* |  | -0.838 | 0.305 | 1.248 |
| Age | -0.301* | -0.156 | -0.163 |  | -0.237* | -0.184* | -0.174* |
| Resting heart rate | -0.043* | -0.040* | -0.037* |  | -0.057* | -0.054* | -0.057* |
| Physical activity index | 0.143* | 0.131* | 0.138* |  | 0.116* | 0.116* | 0.117* |
| Body-fat percentage | -0.480* | -0.507* | -0.565* |  | -0.392* | -0.403* | -0.416* |
| Heart disease | -2.024* | -1.641* | -1.810* |  | -1.223* | -1.382* | -1.417* |
| Current smoking | -3.384* | -2.196* | -2.209* |  | -2.124* | -1.691* | -1.604* |
| R^2^ | 0.4400 | 0.4566 | 0.4473 |  | 0.4671 | 0.4771 | 0.4837 |
| Adjusted R^2^ | 0.4330 | 0.4497 | 0.4402 |  | 0.4603 | 0.4702 | 0.4770 |
| Change-point p-value† | 0.0031 | 0.0006 | 0.2211 |  | 0.0041 | 0.1846 | 0.1704 |
| Definition of abbreviations: VO_2peak_ = peak oxygen uptake; FEV_1_ = forced expiratory volume in 1 second; D_LCO_ = diffusing capacity of the lung for carbon monoxide; D_LCO_/VA = D_LCO_ corrected for estimated alveolar volume; R^2^ = coefficient of determination. Table showing hockey-stick regression models with VO_2peak_ as dependent variable. The variable with an added change-point is given in column heading. Left slope = coefficient for variable up to change-point. Right slope = coefficient for variable after change-point. *P-value<0.05. †P-value for structural change at identified change-point (Chow test). | | | | | | | |

| Additional table 2. Multiple linear regression models with VO_2peak_ (ml/min/kg) as dependent variable | | | | | | | |
| --- | --- | --- | --- | --- | --- | --- | --- |
|  | Men | | |  | Women | | |
|  | FEV_1_ | D_LCO_ | D_LCO_/VA |  | FEV_1_ | D_LCO_ | D_LCO_/VA |
| Constant | 57.36* | 49.56* | 49.92* |  | 58.82* | 53.62* | 51.90* |
| Variable | 2.073* | 0.868* | 6.730* |  | 0.874* | 0.466* | 3.433* |
| Age | -0.251* | -0.156 | -0.160 |  | -0.244* | -0.185* | -0.177* |
| Resting heart rate | -0.045* | -0.037* | -0.037* |  | -0.060* | -0.057* | -0.058* |
| Physical activity index | 0.143* | -0.131* | 0.138* |  | 0.114* | 0.114* | 0.116* |
| Body-fat percentage | -0.474* | -0.511* | -0.567* |  | -0.392* | -0.404* | -0.418* |
| Heart disease | -2.179* | -1.730* | -1.803* |  | -1.358* | -1.401* | -1.453* |
| Current smoking | -3.582* | -2.370* | -2.288* |  | -2.154* | -1.831* | -1.712* |
| R^2^ | 0.4285 | 0.4452 | 0.4461 |  | 0.4569 | 0.4742 | 0.4813 |
| Adjusted R^2^ | 0.4230 | 0.4396 | 0.4406 |  | 0.4515 | 0.4689 | 0.4761 |
| Sig vs. basic model | 0.0000 | 0.0000 | 0.0000 |  | 0.0253 | 0.0003 | 0.0000 |
| Definition of abbreviations: VO_2peak_ = peak oxygen uptake; FEV_1_ = forced expiratory volume in 1 second; D_LCO_ = diffusing capacity of the lung for carbon monoxide; D_LCO_/VA = D_LCO_ corrected for estimated alveolar volume; R^2^ = coefficient of determination. Table showing multiple linear regression models with VO_2peak_ as dependent variable. Variable = beta coefficient for the variable given in column heading. *P-value<0.05. Adjusted R^2^ for VO_2peak_ in a basic linear model without pulmonary function measurements, including only age, physical activity index, resting heart rate, body-fat percentage, smoking status and heart disease history were 0.393 for men and 0.448 for women. Sig vs. basic model = significance level of R^2^-change from basic linear model (f-test). | | | | | | | |

| Additional table 3. Curvilinear regression models with VO_2peak_ (ml/min/kg) as dependent variable | | | | | | | | |
| --- | --- | --- | --- | --- | --- | --- | --- | --- |
|  | Men | | |  | | Women | | |
|  | FEV_1_ | D_LCO_ | D_LCO_/VA | |  | FEV_1_ | D_LCO_ | D_LCO_/VA |
| Constant | 50.75* | 34.70* | 44.52* |  | | 49.07* | 47.47* | 46.67* |
| Variable | 7.830* | 4.238* | 15.02 |  | | 8.962* | 2.141* | 10.86* |
| Variable^2^ | -0.949* | -0.183* | -3.034 |  | | -1.785* | -0.123 | -2.658 |
| Age | -0.275* | -0.156 | -0.163 |  | | -0.234* | -0.179* | -0.176* |
| Resting heart rate | -0.043* | -0.040* | -0.037* |  | | -0.058* | -0.055* | -0.057* |
| Physical activity index | 0.142* | 0.132* | 0.138* |  | | 0.115* | 0.115* | 0.116* |
| Body-fat percentage | -0.480* | -0.507* | -0.565* |  | | -0.392* | -0.403* | -0.417* |
| Heart disease | -2.061* | -1.656* | -1.808* |  | | -1.234* | -1.404* | -1.448* |
| Current smoking | -3.379* | -2.182* | -2.213* |  | | -2.158* | -1.711* | -1.635* |
| R^2^ | 0.4350 | 0.4572 | 0.4470 |  | | 0.4625 | 0.4765 | 0.4826 |
| Adjusted R2 | 0.4287 | 0.4511 | 0.4407 |  | | 0.4564 | 0.4704 | 0.4766 |
| P-value† | 0.0042 | 0.0001 | 0.2918 |  | | 0.0068 | 0.0857 | 0.1918 |
| Definition of abbreviations: VO_2peak_ = peak oxygen uptake; FEV_1_ = forced expiratory volume in 1 second; D_LCO_ = diffusing capacity of the lung for carbon monoxide; D_LCO_/VA = D_LCO_ corrected for estimated alveolar volume; R^2^ = coefficient of determination. Table showing curvilinear models with VO_2peak_ as dependent variable. Variable with squared variable given in column heading. *P-value<0.05. †P-value for R^2^-increase from added squared variable (f-test). | | | | | | | | |
